# Cytotoxic phenazine and antiallergic phenoxazine alkaloids from an arctic *Nocardiopsis dassonvillei* SCSIO 502F

**DOI:** 10.1007/s13659-023-00408-w

**Published:** 2023-10-18

**Authors:** Yue Song, Qi-Yang Li, Meng-Jing Cong, Xiao-Yan Pang, Bo Chen, Yong-Hong Liu, Li Liao, Jun-Feng Wang

**Affiliations:** 1 CAS Key Laboratory of Tropical Marine Bio-Resources and Ecology/Guangdong Key Laboratory of Marine, Materia Medica/Innovation Academy of South China Sea Ecology and Environmental Engineering, South China Sea Institute of Oceanology, Chinese Academy of Sciences, Guangzhou, 510301 China; 2 Key Laboratory for Polar Science, MNR, Polar Research Institute of China, Shanghai, 200136 China; 3 Department of Pharmacology and Therapeutics, McGill University, Montreal, H3A 0G4 Canada; 4 University of Chinese Academy of Sciences, 19 Yuquan Road, Beijing, 100049 China; 5 Sanya Institute of Marine Ecology and Engineering, Sanya, 572000 China; 6 School of Oceanography, Shanghai Jiao Tong University, Shanghai, 200240 China

**Keywords:** *Nocardiopsis dassonvillei*, Arctic Ocean, Phenazine, Phenoxazine, Cytotoxic, Antiallergic

## Abstract

Microbes well-adapted to the Arctic Ocean are promising for producing novel compounds, due to their fancy strategies for adaptation and being under-investigated. Two new phenazine alkaloids (**1** and **2**) and one new phenoxazine (**3**) were isolated from *Nocardiopsis dassonvillei* 502F, a strain originally isolated from Arctic deep-sea sediments. AntiSMASH analysis of the genome of *Nocardiopsis dassonvillei* 502F revealed the presence of 16 putative biosynthetic gene clusters (BGCs), including a phenazine BGC. Most of the isolated compounds were evaluated for their antibacterial, antiallergic, and cytotoxic activities. Among them, compounds **4** and **5** exhibited potent in vitro cytotoxic activities against osteosarcoma cell line 143B with IC_50_ values 0.16 and 20.0 μM, respectively. Besides, the results of antiallergic activities of compounds **6**–**8** exhibited inhibitory activities with IC_50_ values of 10.88 ± 3.05, 38.88 ± 3.29, and 2.44 ± 0.17 μg/mL, respectively (IC_50_ 91.6 μM for the positive control loratadine).

**Graphical Abstract:**

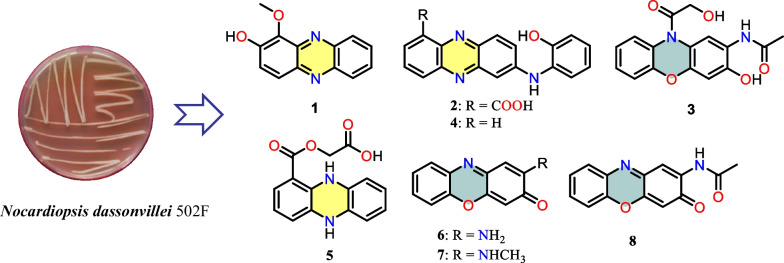

**Supplementary Information:**

The online version contains supplementary material available at 10.1007/s13659-023-00408-w.

## Introduction

Phenazines and phenoxazines consist of a large group of nitrogen-containing heterocyclic compounds that differ in their chemical and physical properties depending on the type and location of the functional groups present [1, 2]. More than 100 different phenazine structural derivatives have been found in nature. Microbes inhabiting the extreme environments in the polar regions are attracting increasing attention in ecology and biotechnology. The polar regions are generally characterized by permanently low temperature, high pressure, high salinity, and extreme gradients of nutrients and light etc. To survive and propagate in such harsh environments, microbes have developed various strategies including producing novel bioactive compounds [3]. Therefore, polar microorganisms are promising sources for novel natural products or chemical scaffolds with pharmaceutically relevant biological activity, considering their diversity and novelty. Previous studies have shown great potential of polar microorganisms in producing natural products with various bioactivities, including antimicrobial [4], anticancer [5], and immunosuppressive activities etc. [6] (as summarized in [7] and [8]). More recently, genome-based analyses have revealed biosynthetic potential of microbes from the polar regions, especially rare Actinobacteria groups (i.e., non-*Streptomyces* Actinobacteria) [9–11]. Much greater biosynthetic potential has been indicated by genomic data mining than previous investigation based merely on traditional isolation.

**Fig. 1:**
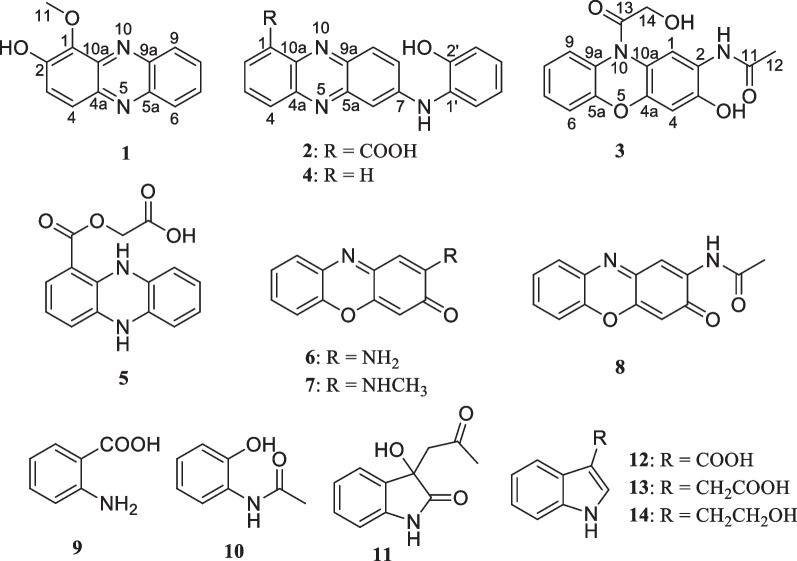
Chemical structures of compounds **1**‒**14**

Previously, strain 502F was isolated from Arctic deep sediments and showed antimicrobial activity [10]. Primary analysis based on genome sequence indicated biosynthetic potential of various secondary metabolites. As part of our continuing efforts to explore the chemical diversity of polar microorganisms for drug discovery, strain 502F was selected for chemical investigative. Multiple-step chromatographic isolation of the crude extracts afforded two new phenazine alkaloids (**1** and **2**) and one new phenoxazine (**3**), as well as the previously reported compounds, *N*-(2-hydroxyphenyl)-2-phenazinamine (**4**) [4], endophenazine (**5**) [12], 2-aminophenoxazin-3-one (**6**) [4], 2-(*N*-methylamino)-3-phenoxazone (**7**) [4], *N*-acetylquestiomycin A (**8**) [13], 2-aminobenzoic acid (**9**) [14], 2-(acetylamino)-phenol (**10**) [15], 3-(2-ox-opropyl)-3-hydroxyind-olin-2-one (**11**) [16], indolyl-3-carboxylic acid (**12**) [17], indole-3-acetic acid (**13**) [18], and tryptophol (**14**) [19]. Herein, details of the isolation, structural elucidation, and antibacterial, antiallergic, and cytotoxic activities of compounds (**1**–**14**) are described (Fig. 1).

## Results and discussion

**Fig. 2:**
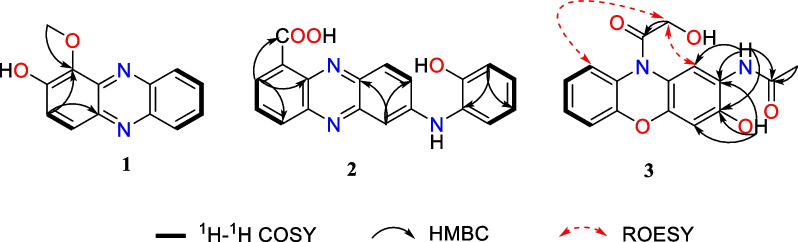
The key ^1^H-^1^H COSY, HMBC, and ROESY correlations of **1**–**3**

Compound **1** was obtained as a yellow solid. The molecular formula C_13_H_10_N_2_O_2_ was established upon analysis of the HRESIMS peak at *m/z* 227.0815 [M + H]^+^, indicating 10 degrees of unsaturation. The UV absorptions at 233, 274, 371, and 433 nm implied the presence of an extended conjugated system. The ^1^H and ^13^C NMR spectroscopic data suggested the presence of one methoxy group, six methine, and six quaternary carbons. The ^1^H NMR spectroscopic data of **1** (Table 1) revealed six aromatic protons at [*δ*_H_ 7.67 (1H, d, *J* = 9.4 Hz, H-3), 7.93 (1H, d, *J* = 9.4 Hz, H-4), and at *δ*_H_ 8.17 (1H, d, *J* = 8.6 Hz, H-6), 7.84 (1H, ddd, *J* = 8.5, 6.6, 1.3 Hz, H-7), 7.90 (1H, ddd, *J* = 8.5, 6.6, 1.3 Hz, H-8), 8.25 (1H, d, *J* = 8.6 Hz, H-9)], which were assigned to be the typical AB and AA′BB′ spin systems, respectively, with the aid of the COSY correlations of H-3/H-4 and H-6/H-7/H-8/H-9 (Fig. 2). These indicated that **1** had a 1,2- or 1,4-disubstituted phenazine moiety. In the HMBC spectrum (Fig. 2), the key correlations of H-3 with C-1 and C-4a, and H-4 with C-2 and C-10a, confirmed that **1** was a 1,2-disubstituted phenazine. Combining the molecular formula and the NMR data above, the remaining one methoxy and one hydroxy were required in compound **1**. Dihydroxylation by the corresponding -OCH_3_ and -OH groups in **1**, located at C-1 and C-2, respectively, were further supported by the HMBC correlations of H_3_-11 (*δ*_H_ 4.14) with C-1 and chemical shift of C-2 (*δ*_C_ 152.1) (Fig. 2). Hence, the structure of **1** was identified as 1-methoxyphenazin-2-ol.

**Table 1:** ^1^H and ^13^C NMR Data for **1**–**3** (700, 175 MHz, TMS, *δ* ppm)

No	**1**^**a**^		**2**^**a**^		**3**^**b**^	
	*δ*_C_	*δ*_H_ (*J* in Hz)	*δ*_C_	*δ*_H_ (*J* in Hz)	*δ*_C_	*δ*_H_ (*J* in Hz)
1	138.6, C		126.0, C		118.8, C	7.88, s
2	152.1, C		134.4, CH	8.61, d (7.6)	121.8, C	
3	127.8, CH	7.67, d (9.4)	131.2, CH	7.96, dd (8.6, 7.1)	147.3, C	
4	126.6, CH	7.93, d (9.4)	134.4, CH	8.27, d (7.6)	103.4, CH	6.70, s
4a	141.5, C		144.5, C		146.9, C	
5a	142.3, C		150.1, C		150.3, C	
6	130.1, CH	8.17, d (8.6)	102.2, CH	7.21, d (2.4)	116.6, CH	7.20, dd (8.1, 1.3)
7	130.7, CH	7.84, ddd (8.5, 6.6, 1.3)	147.7, C		127.1, CH	7.26, td (8.3, 1.4)
8	132.2, CH	7.90, ddd (8.5, 6.6, 1.3)	131.1, CH	7.86, dd (9.3, 2.4)	123.5, CH	7.17, td (7.8, 1.3)
9	129.5, CH	8.25, d (8.6)	129.7, CH	8.11, d (9.3)	125.0, CH	7.58, dd (7.9, 1.4)
9a	129.5, C		138.8, C		128.7, C	
10a	140.6, C		138.6, C		*	
11	62.1, CH_3_	4.14, s	168.9, C		168.8, C	
12					23.5, CH_3_	2.08, s
13					171.1, C	
14					60.4, CH_2_	4.28, s
1′			128.4, C			
2′			152.5, C			
3′			117.3, CH	7.00, dd (8.1, 1.4)		
4′			127.5, CH	7.13, td (8.1, 1.5)		
5′			120.9, CH	6.95, td (7.6, 1.4)		
6′			125.7, CH	7.43, dd (7.8, 1.5)		
2-NH						9.32, s
3-OH						10.26, s

^a^Data were recorded in CD_3_OD. ^b^Data were recorded in DMSO-*d*_6_. *Not observed

Compound **2** was isolated as dark purple powder with the molecular formula C_19_H_13_N_3_O_3_ as determined by HRESIMS indicating 15 degrees of unsaturation. The ^1^H NMR spectrum showed characteristic signals for one typical 1,2,3-substituted benzene system [*δ*_H_ 8.61 (d, *J* = 7.6 Hz, H-2), 7.96 (dd, *J* = 8.6, 7.1 Hz, H-3), and 8.27 (d, *J* = 7.6 Hz, H-4)], one ABX spin system [*δ*_H_ 7.21 (d, *J* = 2.4 Hz, H-6), 7.86 (dd,* J* = 9.3, 2.4 Hz, H-8), and 8.11 (d, *J* = 9.3 Hz, H-9)], and one AA'BB' spin system [*δ*_H_ 7.00 (dd, *J* = 8.1,1.4 Hz, H-3′), 7.13 (td, *J* = 8.1, 1.5 Hz, H-4′), 6.95 (td, *J* = 7.6, 1.4 Hz, H-5′), and 7.43 (dd, *J* = 7.8,1.5 Hz, H-6′)], respectively. The ^1^H-^1^H COSY correlation of H-2/H-3/H-4, H-8/H-9, and H-3′/H-4′/H-5′/H-6′ displayed the key spin systems. Careful comparison of ^1^H and ^13^C NMR data with those of *N*-(2-hydroxyphenyl)-2-phenazinamine (**4**), revealed a high degree of similarity between them, except for the presence of carboxyl group (C-11) instead of the corresponding aromatic proton (H-1), which was confirmed by the key HMBC correlations from H-2 to C-4, C-10a, and C-11 (COOH) (Fig. 2). To further define the structure of **2**, the ^13^C NMR spectra of** 2**–**1** (carboxylic group located at C-1) and** 2**–**2** (carboxylic group located at C-4) were calculated using the gauge invariant atomic orbitals (GIAO) method at the B3LYP/6-31G(d,p)/PCM (Methanol) (Additional file 1: Tables S2–S4). Subsequent analyses of the calculated ^13^C NMR values led to a deduction that** 2**–**1** adopted the suitable structure with a DP4 + probability of 100%, which allowed the assignment of the carboxylic group located at C-1 in compound **2**. Therefore, the structure of **2** was identified as *N*-(2-hydroxyphenyl)-2-phenazinamine-1-carboxylic acid.

Compound **3** was obtained as a yellow solid and had a molecular formula of C_16_H_14_N_2_O_5_ as determined by HRESIMS (*m/z* 315.0973 [M + H]^+^), requiring 11 degrees of unsaturation. The ^1^H NMR spectrum (Table 1) and COSY correlations exhibited the resonances for eight aromatic protons, of which two singlets at [*δ*_H_ 7.88 (1H, s, H-1) and 6.70 (1H, s, H-4)], one AA'BB' spin system at [*δ*_H_ 7.20 (dd, *J* = 8.1,1.3 Hz, H-6), 7.26 (td, *J* = 8.3, 1.4 Hz, H-7), 7.17 (td, *J* = 7.8, 1.3 Hz, H-8), and 7.58 (dd, *J* = 7.9, 1.4 Hz, H-9)] were observed. These findings in association with ^13^C NMR data (Table 1) identified 12 aromatic resonances consistent with the molecule containing two aromatic rings. The HMBC correlations from 2-NH (*δ*_H_ 7.91) to C-1 (*δ*_C_ 118.8), C-2 (*δ*_C_ 121.8), and C-3 (*δ*_C_ 147.3) allowed it to be positioned at C-2. Further HMBC correlations from 2-NH and H_3_-12 (*δ*_H_ 2.08) to C-11 (*δ*_C_ 168.8) allowed for an acetyl moiety to be located at the nitrogen. The chemical shift of C-3 (*δ*_C_ 147.3) indicated oxygenation at this site. Besides, the exchangeable proton 3-OH (*δ*_H_ 10.26) in turn possessed HMBC correlations to C-2, C-3, and C-4 (*δ*_C_ 103.4) confirmed the –OH group to be located at C-3. The oxymethylene H_2_-14 (*δ*_H_ 4.28) demonstrated a HMBC correlation to C-13 (*δ*_C_ 171.1), allowing for a hydroxyacetyl moiety to be constructed. This part has no further relevance in any 2D NMR experiments, suggesting that it is attached to the molecule by heteroatoms. Due to the presence of 2-NH and 3-OH, there was only one position for the hydroxyacetyl fragment located at 10-N, which was also consistent with the degrees of unsaturation and molecular formula. Furthermore, this deduction was supported by the ROESY correlations of H_2_-14 (*δ*_H_ 4.28) with H-1 (*δ*_H_ 7.88) and H-9 (*δ*_H_ 7.58) (Fig. 2). Consequently, the structure of **3** was determined to be *N*-(3-hydroxy-10-(2-hydroxyacetyl)-10*H*-phenoxazin-2-yl)acetamide.

Phenazine and phenoxazine-based alkaloids are a large group of structurally unique natural products containing a tricyclic core consist of two nitrogen atoms or nitrogen and oxygen atoms, respectively. Since 1859, when Fordos reported the isolation of the blue pigment known as pyocyanin (5-*N*-methylphenazine-1-one), more than 100 different natural phenazines have been identified. Although phenoxazinone-type alkaloids are also widely distributed in nature, alkaloids with benzoxazine nuclei are relatively rare compared to phenazines. Furthermore, phenoxazinone-type compound **3** containing carbon substitution at N-10 is the third reported in nature. Naturally occurring phenazine and phenoxazine-based alkaloids have received much attention due to their interesting biological activities, including anti-bacterial, anti-cancer, anti-parasitic.

**Fig. 3:**
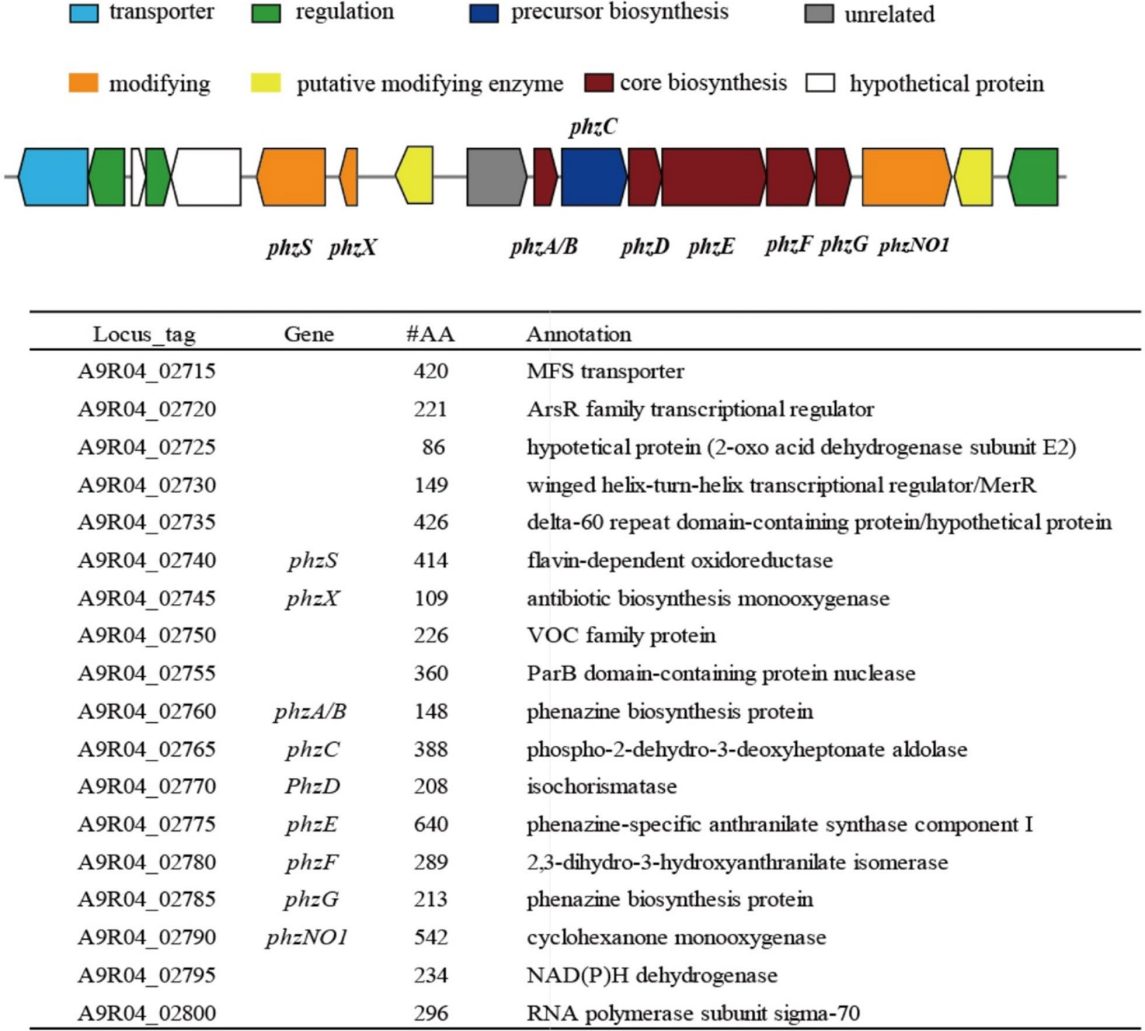
Putative biosynthetic gene cluster of phenazines predicted in the genome of strain 502F

AntiSMASH analysis of the genome of *Nocardiopsis dassonvillei* 502F revealed the presence of 16 putative biosynthetic gene clusters (BGCs) (Additional file 1: Table S1). The BGCs contained quite diverse types, including phenazine, ectoine, PKS, NRPS, and small RPS etc. Most of the remaining BGCs shared quite low identities with known clusters, indicating their potential in producing new compounds. A putative phenazine biosynthetic gene cluster was predicted in the genome, containing the core biosynthetic genes (phzA/B, phzD, phzE, phzF, phzG and phzNO1), possible modifying genes (phzS, phzX, phzNO1, phzO and a VOC family protein-encoding gene), transporter and transcription regulation genes (Fig. 3). The core biosynthetic genes were responsible for conversion of the precursor chorismate to phenazine-1-carboxylic acid (PCA) or phenazine-1,6-dicarboxylic acid (PDC). PCA or PDC are two precursors that can be modified further to create more complex phenazine derivatives, resulting in a serial of phenazines such as compounds **1**, **2**, **4**, and **5**.

Most of the isolated compounds were evaluated for their antibacterial, antiallergic, and cytotoxic activities. Compounds (**1**–**14**) were evaluated in 96-well microtiter plates using a modification of the broth microdilution method. The indicator bacteria strains included three Gram-positive bacteria, *methicillin-resistant Staphyloccocus aureus* (MRSA), *Staphyloccocus aureus* (ATCC 29213), *Enterococcus faecalis* (ATCC 29212), respectively [20]. However, no compounds showed potent antibacterial activities. Cytotoxic activities (C4-2B, MB-231, 143B, MGC803, and A549 cells) were evaluated using the CCK-8 method as described previously [21]. Among them, compounds **4** and **5** exhibited potent in vitro cytotoxic activities against osteosarcoma cell line 143B with IC_50_ values 0.16 and 20.0 μM, respectively. The antiallergic activity was measured for the efficiency of the RBL-2H3 cell degranulation inhibition rate using an IgE-mediated mast cell allergic reaction [22]. The results of antiallergic activity of compounds **6**–**8** showed obvious inhibition rates (86.35 ± 3.11%, 38.02 ± 3.98%, and 92.84 ± 9.37% inhibition at the concentration of 20 μg/mL) with IC_50_ values of 10.88 ± 3.05, 38.88 ± 3.29, and 2.44 ± 0.17 μg/mL (Table 2), respectively, while loratadine was used as positive control with IC_50_ value of 91.6 μM.

**Table 2:** Antiallergic activities of compounds **1**–**8** (IC_50_, *μ*M)

1	2	3	4	5	6	7	8	Loratadine
> 100	> 100	> 100	> 100	> 100	10.88 ± 3.05	38.88 ± 3.29	2.44 ± 0.17	91.6

Loratadine as the positive control

### Supplementary Information

**Additional file 1.** Supplementary data.

## Data Availability

All relevant data are within the manuscript and its Additional files.
