# Supplementary material for: Cytotoxic phenazine and antiallergic phenoxazine alkaloids from an arctic Nocardiopsis dassonvillei SCSIO 502F
Source: Nat Prod Bioprospect. 2023 Oct 18;13(1):41. doi: 10.1007/s13659-023-00408-w (PMC10581944; doi:10.1007/s13659-023-00408-w)
Supplement: Supplementary file 1 — Additional file 1. Supplementary data. [file 13659_2023_408_MOESM1_ESM.doc]

**Supplementary data**

**Cytotoxic phenazine and antiallergic phenoxazine alkaloids from an arctic *Nocardiopsis dassonvillei* SCSIO 502F**

Yue Song^1,4^, Qi-Yang Li^1,3^, Meng-Jing Cong^1,4^, Xiao-Yan Pang^1^, Bo Chen^2^,

Yong-Hong Liu^1,4,5^, Li Liao^2,6*^, Jun-Feng Wang^1,4,5*^

**Affiliation**

^1^ CAS Key Laboratory of Tropical Marine Bio-resources and Ecology/Guangdong Key Laboratory of Marine Materia Medica/Innovation Academy of South China Sea Ecology and Environmental Engineering, South China Sea Institute of Oceanology, Chinese Academy of Sciences, Guangzhou 510301, China

^2^ Key Laboratory for Polar Science, MNR, Polar Research Institute of China, Shanghai, 200136, PR China

^3^ Department of Pharmacology and Therapeutics, McGill University, Montreal H3A 0G4, Canada

^4^ University of Chinese Academy of Sciences, 19 Yuquan Road, Beijing 100049, China

^5^ Sanya Institute of Marine Ecology and Engineering, Sanya 572000, China

^6^ School of Oceanography, Shanghai Jiao Tong University, Shanghai 200240, China

**List of supporting information Page**

The 16s rRNA sequence of *Nocardiopsis dassonvillei* SCSIO 502F……………………………...……S3

**Table S1**. Biosynthetic gene clusters predicted by antiSMASH program...........….................................S8

**Table S2.** Energies of dominative conformers of **2** at MMFF force field...….........................................S8

**Table S3** Energies of dominative conformers at B3LYP/6-31G(d,p) in methanol...................................S9

**Table S4** DP4+ analysis of calculated ^13^C-NMR data of **2**...…................................................................S10

**Figure S1**. The ^1^H NMR spectrum of compound **1** in CD_3_OD……………………………….....…...S11

**Figure S2**. The ^13^C NMR spectrum of compound **1** in CD_3_OD….……………………………......…S11

**Figure S3**. The HMQC spectrum of compound **1** inCD_3_OD……………………...............……...…S12

**Figure S4**. The HMQC expanded regions of compound **1** in CD_3_OD……………...............……...…S12

**Figure S5**. The ^1^H-^1^H COSY spectrum of compound **1** in CD_3_OD……………………...........…...S13

**Figure S6**. The ^1^H-^1^H COSY expanded regions of compound **1** in CD_3_OD……...............……...…S13

**Figure S7**. The HMBC spectrum of compound **1** in CD_3_OD……………………….………………S14

**Figure S8**. The HMBC expanded regions of compound **1** in CD_3_OD……………...............……...…S14

**Figure S9**. The HRESIMS spectrum of compound **1**……..…………………………..............…..S15

**Figure S10**. The UV spectrum of compound **1**…….........…………………………..............…..S15

**Figure S11**. The ^1^H NMR spectrum of compound **2** in CD_3_OD…………………………….....…...S16

**Figure S12**. The ^13^C NMR spectrum of compound **2** in CD_3_OD….…………………………......…S16

**Figure S13**. The HMQC spectrum of compound **2** inCD_3_OD……………………...............……...…S17

**Figure S14**. The HMQC expanded regions of compound **2** in CD_3_OD…………...............……...…S17

**Figure S15**. The ^1^H-^1^H COSY spectrum of compound **2** in CD_3_OD……………………...........…...S18

**Figure S16**. The ^1^H-^1^H COSY expanded regions of compound **2** in CD_3_OD……...............……...…S18

**Figure S17**. The HMBC spectrum of compound **2** in CD_3_OD……………………….………………S19

**Figure S18**. The HMBC expanded regions of compound **2** in CD_3_OD…………...............……...…S19

**Figure S19**. The HRESIMS spectrum of compound **2**……..…………………………..............…..S20

**Figure S20**. The UV spectrum of compound **2**…….........…………………………..............…..S20

**Figure S21**. The ^1^H NMR spectrum of compound **3** in DMSO-*d*_6_…………………………….....…...S21

**Figure S22**. The ^13^C NMR spectrum of compound **3** in DMSO-*d*_6_….…………………………......…S21

**Figure S23**. The HMQC spectrum of compound **3** in DMSO-*d*_6_…………………...............……...…S22

**Figure S24**. The ^1^H-^1^H COSY spectrum of compound **3** in DMSO-*d*_6_……………………...........…...S22

**Figure S25**. The HMBC spectrum of compound **3** in DMSO-*d*_6_……………………….………………S23

**Figure S26**. The ROESY spectrum of compound **3** in DMSO-*d*_6_……………………….………………S23

**Figure S27**. The HRESIMS spectrum of compound **3**……..…………………………..............…..S24

**Figure S28**. The UV spectrum of compound **3**…….........…………………………..............…..S24

**The 16s rRNA sequence of *Nocardiopsis dassonvillei* 502F** TTTATGGAGAGTTTGATCCTGGCTCAGGACGAACGCTGGCGGCGTGCTTAACACATGCAAGTCGAGCGGTAAGGCCCTTCGGGGTACACGAGCGGCGAACGGGTGAGTAACACGTGAGCAACCTGCCCCTGACTCTGGGATAAGCGGTGGAAACGCCGTCTAATACCGGATACGACCCGCCACCTCATGGTGGAGGGTGGAAAGTTTTTCGGTCAGGGATGGGCTCGCGGCCTATCAGCTTGTTGGTGGGGTAACGGCCTACCAAGGCGATTACGGGTAGCCGGCCTGAGAGGGCGACCGGCCACACTGGGACTGAGACACGGCCCAGACTCCTGCGGGAGGCAGCAGTGGGGAATATTGCGCAATGGGCGAAAGCCTGACGCAGCGACGCCGCGTGGGGGATGACGGCCTTCGGGTTGTAAACCTCTTTTACCACCAACGCAGGCTTCCAGTTCTCTGGAGGTTGACGGTAGGTGGGGAATAAGGACCGGCTAACTACGTGCCAGCAGCCGCGGTAATACGTAGGGTCCGAGCGTTGTCCGGAATTATTGGGCGTAAAGAGCTCGTAGGCGGCGTGTCGCGTCTGCTGTGAAAGACCGGGGCTTAACTCCGGTTCTGCAGTGGATACGGGCATGCTAGAGGTAGGTAGGGGAGACTGGAATTCCTGGTGTAGCGGTGAAATGCGCAGATATCAGGAGGAACACCGGTGGCGAAGGCGGGTCTCTGGGCCTTACCTGACGCTGAGGAGCGAAAGCATGGGGAGCGAACAGGATTAGATACCCTGGTAGTCCATGCCGTAAACGTTGGGCGCTAGGTGTGGGGACTTTCCACGGTTTCCGCGCCGTAGCTAACGCATTAAGCGCCCCGCCTGGGGAGTACGGCCGCAAGGCTAAAACTCAAAGGAATTGACGGGGGCCCGCACAAGCGGCGGAGCATGTTGCTTAATTCGACGCAACGCGAAGAACCTTACCAAGGTTTGACATCACCCGTGGACTCGCAGAGATGTGAGGTCATTTAGTTGGCGGGTGACAGGTGGTGCATGGCTGTCGTCAGCTCGTGTCGTGAGATGTTGGGTTAAGTCCCGCAACGAGCGCAACCCTTGTTCCATGTTGCCAGCACGTAATGGTGGGGACTCATGGGAGACTGCCGGGGTCAACTCGGAGGAAGGTGGGGATGACGTCAAGTCATCATGCCCCTTATGTCTTGGGCTGCAAACATGCTACAATGGCCGGTACAATGGGCGTGCGATACCGTAAGGTGGAGCGAATCCCTAAAAGCCGGTCTCAGTTCGGATTGGGGTCTGCAACTCGACCCCATGAAGGTGGAGTCGCTAGTAATCGCGGATCAGCAACGCCGCGGTGAATACGTTCCCGGGCCTTGTACACACCGCCCGTCACGTCATGAAAGTCGGCAACACCCGAAACTTGCGGCCTAACCCCTTGTGGGAGGGAGTGAGTGAAGGTGGGGCTGGCGATTGGGACGAAGTCGTAACAAGGTAGCCGTACCGGAAGGTGCGGCTGGATCACCTCCTTTCTAAGGAG

**Experimental**

*1. General experimental procedures*

UV spectra were recorded on a UV-2600 spectrometer (Shimadzu). The 1D and 2D NMR spectra were collected using a Bruker AV-700 MHz NMR spectrometer (Bruker) at 700MHz for ^1^H NMR and 175 MHz for ^13^C NMR with tetramethylsilane (TMS) as the internal standard. HRESIMS and ESIMS spectra data were recorded on a MaXis quadrupole-time-of-flight mass spectrometer and an amaZon SL ion trap mass spectrometer (Bruker), respectively. Vacuum-liquid chromatography (VLC) used silica gel H (Qingdao Marine Chemical Factory). Thin layer chromatography (TLC) and column chromatography (CC) were performed on over silica gel (200–300 mesh) (Qingdao Marine Chemical Factory) and plates precoated with silica gel GF254 (10–40 *μ*m), C_18_ reversed phase silica gel (RP-18, 150 to 200 mesh, Fuji Silysia Chemical Ltd., Japan), and Sephadex LH-20 (Amersham Biosciences, Sweden), respectively. Compounds were detected with an Angilent 1260 HPLC apparatus using an ODS column (YMC-pack ODS-A, 10 × 250 mm, 5 *μ*m, 4 mL/min). All solvents used were of analytical grade (Tianjin Fuyu Chemical and Industry Factory).

*2. Actinomycete Material*

The actinomycete strain, *Nocardiopsis* sp. 502F, was isolated from deep-sea sediments over 2000 m below sea level in the Arctic Ocean (75°00.507′N 162°01.744′W). Since the strain has identical 16S rRNA gene with *Nocardiopsis dassonvillei* IMRU 509^T^, it is tentatively classified as *Nocardiopsis dassonvillei* 502F.

*3. Fermentation and Extraction*

Strain 502F was inoculated in 1000 mL × 266 conical flasks containing the liquid medium (300 mL/flask) composed of soluble starch (25 g/L), soybean powder (20 g/L), MgSO_4_·7H_2_O (0.6 g/L), CaCO_3_ (0.2 g/L) and sea salt (30 g/L) (adjusted pH to 7.0 before sterilization) for 9 days at 24 °C on a rotary shaker at 180 rpm. The fermented whole broth (80 L) was filtered through cheesecloth to separate into filtrate and mycelia. The filtrate was concentrated under vacuum to about a quarter of the original volume and then extracted three times with EtOAc, while the mycelia were extracted three times with acetone. The acetone solution was evaporated under reduced pressure to afford an aqueous solution. The EtOAc solution was extracted by EtOAc three times to obtain another EtOAc solution. Three EtOAc solutions were mixed and concentrated under reduced pressure to obtain a dark brown gum (21.6 g).

*4. Purification*

The crude extract (21.6 g) was subjected to vaccum liquid chromatography (VLC) on a silica gel column using step gradient elution with MeOH–CH_2_Cl_2_ (0–100%) to separate into nine fractions based on TLC properties. Fraction 1 (3.2 g) was separated into four subfractions (Frs.1-1–1-4) by RP-18 silica gel eluting with MeOH-H_2_O (30%–100%). Fr.1-1 was directly separated by HPLC (25% MeOH-H_2_O) to yield **10** (6.0 mg, *t*_R_ 22.3 min). Fr.1-4 was then separated by HPLC (60% MeOH-H_2_O + 0.1% CF_3_CO_2_H) to yield **9** (4.3 mg, *t*_R_ 7.9 min), **6** (4.7 mg, *t*_R_ 16.3 min), **7** (3.7 mg, *t*_R_ 17.8 min), and **8** (4.1 mg, *t*_R_ 20.4 min), respectively. Fraction 2 (2.6 g) was separated into five subfractions (Frs.2-1–2-5) by RP-18 silica gel eluting with MeOH-H_2_O (20%–100%). Fr.2-2 was divided into four parts (Frs.2-2-1–2-2-4) by HPLC (48% MeOH-H_2_O). Fr.2-2-2 was further purified by HPLC (13% CH_3_CN-H_2_O + 0.1% CF_3_CO_2_H) to yield **14** (7.3 mg, *t*_R_ 54.8 min). Fr.2-2-3 was then purified by HPLC (30% CH_3_CN-H_2_O + 0.1% CF_3_CO_2_H) to yield **1** (3.2 mg, *t*_R_ 16.5 min). Fr.3 (2.9 g) was divided into five parts (Frs.3-1–3-5) by Sephadex LH-20 (MeOH). Fr.3-2 was directly separated by HPLC (21% CH_3_CN-H_2_O + 0.1% CF_3_CO_2_H) to yield **13** (59.0 mg, *t*_R_ 29.4 min). Fr.3-3 was then separated by HPLC (33.5% CH_3_CN-H_2_O + 0.1% CF_3_CO_2_H) to yield **5** (4.0 mg, *t*_R_ 9.2 min), and **12** (1.5 mg, *t*_R_ 13.5 min), respectively. Fraction 4 (3.1 g) was separated into five subfractions (Frs.4-1–4-5) by RP-18 silica gel eluting with MeOH-H_2_O (10%–100%). Fr.4-1 was separated by HPLC (19% MeOH-H_2_O + 0.1% CF_3_CO_2_H) to yield **11** (5.5 mg, *t*_R_ 36.4 min). Fr.4-3 was separated by HPLC (25% CH_3_CN-H_2_O + 0.1% CF_3_CO_2_H) to yield **3** (4.9 mg, *t*_R_ 36.4 min).

*4.1. Compound* ***1***

Brown solid; UV (MeOH) *λ*_max_ (log *ε*): 219 (3.35), 264 (3.85), 362 (2.85) nm; ^1^H NMR and ^13^C NMR data, see Table 1; (+)-HRESIMS *m/z* 227.0815 [M + H]^+^ (calcd for C_13_H_11_N_2_O_2_, 227.0815), 249.0631 [M + Na]^+^ (calcd for C_13_H_10_N_2_NaO_2_, 249.0634).

*4.2. Compound* ***2***

Mulberry solid; UV (MeOH) *λ*_max_ (log *ε*): 206 (4.10), 231 (3.97), 290 (4.02), 495 (3.11) nm; ^1^H NMR and ^13^C NMR data, see Table 1; (+)-HRESIMS *m/z* 332.1027 [M + H]^+^ (calcd for C_19_H_14_N_3_O_3_, 332.1030), 354.0837 [M + Na]^+^ (calcd for C_19_H_13_N_3_NaO_3_, 354.0849), 685.1797 [2M + Na]^+^ (calcd for C_38_H_29_N_6_NaO_6_, 685.1806).

*4.3. Compound* ***3***

Brown solid; UV (MeOH) *λ*_max_ (log *ε*): 215 (4.23), 401 (3.19) nm; ^1^H NMR and ^13^C NMR data, see Table 1; (+)-HRESIMS *m/z* 315.0973 [M + H]^+^ (calcd for C_16_H_15_N_2_O_5_, 315.0975), 337.0800 [M + Na]^+^ (calcd for C_16_H_14_N_2_NaO_5_, 337.0795), 651.1696 [2M + Na]^+^ (calcd for C_32_H_28_N_4_NaO_10_, 651.1698).

5. *Bioinformatic analysis*

The genome sequencing, genome assembly, gene prediction and gene function annotation were performed. Besides, the genome of *Nocardiopsis dassonvillei* 502F was analyzed by AntiSMASH.

*6. ^13^C NMR Calculation Details*

The calculation was performed by using the density functional theory (DFT) as carried out in Gaussian 09. The preliminary conformational distributions search was performed using Spartan 14 the MMFF force field and afforded six and four stable conformers (Table S1) for **2-1** and **2-2**, respectively. To further define the structure of **2**, the ^13^C NMR spectra of **2-1** and **2-2** were calculated using the gauge invariant atomic orbitals (GIAO) method at the B3LYP/6-31G(d,p)/PCM (Methanol) (Tables S2–S4). The calculated ^13^C NMR data of **2-1** (DP4+ probability = 100%) showed a better match with the experimental data of **2** than that of **2-2** (DP4+ probability = 0%), which allowed the assignment of the carboxylic group located at C-1 in compound **2**.


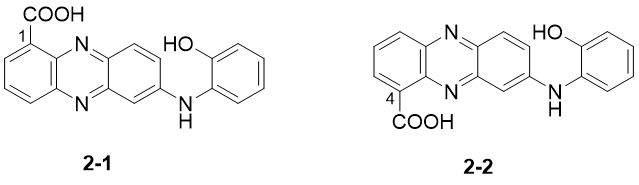


**Table S1.** Biosynthetic gene clusters predicted by antiSMASH program.

| Region | Type | From | To | Length  (Mb) | Most similar known cluster | Similarity % |
| --- | --- | --- | --- | --- | --- | --- |
| Cluster 1 | [phenazine](https://docs.antismash.secondarymetabolites.org/glossary/#phenazine) | 619711 | 640154 | 20.4 | [marinophenazine A / phenaziterpene A](https://mibig.secondarymetabolites.org/go/BGC0001221/1) | 34 |
| Cluster 2 | [ectoine](https://docs.antismash.secondarymetabolites.org/glossary/#ectoine) | 816811 | 827206 | 10.4 | [ectoine](https://mibig.secondarymetabolites.org/go/BGC0000853/1) | 75 |
| Cluster 3 | [T1PKS](https://docs.antismash.secondarymetabolites.org/glossary/#t1pks) | 1348463 | 1406121 | 57.7 | [aureothin](https://mibig.secondarymetabolites.org/go/BGC0000024/1) | 33 |
| Cluster 4 | [NRPS](https://docs.antismash.secondarymetabolites.org/glossary/#nrps) | 2057564 | 2110775 | 53.2 | [coelibactin](https://mibig.secondarymetabolites.org/go/BGC0000324/1) | 90 |
| Cluster 5 | [siderophore](https://docs.antismash.secondarymetabolites.org/glossary/#siderophore) | 2132578 | 2144419 | 11.8 | [desferrioxamine E](https://mibig.secondarymetabolites.org/go/BGC0001478/1) | 100 |
| Cluster 6 | [CDPS](https://docs.antismash.secondarymetabolites.org/glossary/#cdps) | 2225410 | 2246144 | 20.7 | [albonoursin](https://mibig.secondarymetabolites.org/go/BGC0000851/1) | 50 |
| Cluster 7 | [terpene](https://docs.antismash.secondarymetabolites.org/glossary/#terpene) | 2444378 | 2463823 | 19.4 | [leinamycin](https://mibig.secondarymetabolites.org/go/BGC0001101/1) | 2 |
| Cluster 8 | T3PKS,NRPS | 2903138 | 2995399 | 92.3 | [A40926](https://mibig.secondarymetabolites.org/go/BGC0000289/1) | 12 |
| Cluster 9 | [NRPS](https://docs.antismash.secondarymetabolites.org/glossary/#nrps) | 3460055 | 3503586 | 43.5 | [fervenulin](https://mibig.secondarymetabolites.org/go/BGC0001973/1) | 10 |
| Cluster 10 | terpene,bacteriocin | 3922710 | 3945298 | 22.6 | [2-methylisoborneol](https://mibig.secondarymetabolites.org/go/BGC0000657/1) | 75 |
| Cluster 11 | [butyrolactone](https://docs.antismash.secondarymetabolites.org/glossary/#butyrolactone) | 4784628 | 4795731 | 11.1 | [murayaquinone](https://mibig.secondarymetabolites.org/go/BGC0001675/1) | 3 |
| Cluster 12 | [lanthipeptide](https://docs.antismash.secondarymetabolites.org/glossary/#lanthipeptide) | 4889137 | 4912150 | 23.0 | [colicin V](https://mibig.secondarymetabolites.org/go/BGC0001555/1) | 1 |
| Cluster 13 | terpene | 5046463 | 5072222 | 25.8 | isorenieratene | 100 |
| Cluster 14 | T2PKS | 5828857 | 5901333 | 72.5 | allocyclinone | 8 |
| Cluster 15 | NRPS | 5907748 | 5967801 | 60.1 | teicoplanin | 5 |
| Cluster 16 | lanthipeptide,oligosaccharide,other,PKS-like | 6377809 | 6443437 | 65.6 | mycinamicin II | 14 |

**Table S2.** Energies of dominative conformers of **2** at MMFF force field.

| **Configuration** | **Conformer** | **Energy (kcal/mol)** | **Population (%)** |
| --- | --- | --- | --- |
| **2-1** | **2-1a** | 79.93 | 28.7 |
| **2-1** | **2-1b** | 79.97 | 26.9 |
| **2-1** | **2-1c** | 80.20 | 18.0 |
| **2-1** | **2-1d** | 80.47 | 11.5 |
| **2-1** | **2-1e** | 80.69 | 7.9 |
| **2-1** | **2-1f** | 80.76 | 7.0 |
| **2-2** | **2-2g** | 80.04 | 27.1 |
| **2-2** | **2-2h** | 80.17 | 21.9 |
| **2-2** | **2-2i** | 80.22 | 20.1 |
| **2-2** | **2-2j** | 80.75 | 8.3 |

**Table S3** Energies of dominative conformers at B3LYP/6-31G(d,p) in methanol.

| Conformer | Configuration | E (Hartree) | Energy (kcal/mol) | Population (%) |
| --- | --- | --- | --- | --- |
| **2-1a** | **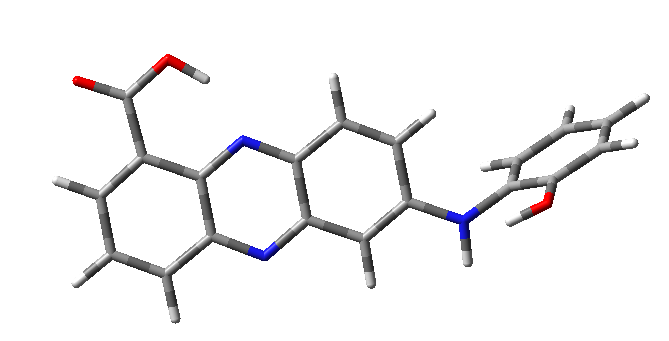** | -1121.59582789 | -703812.59795925 | 13.66 |
| **2-1b** | **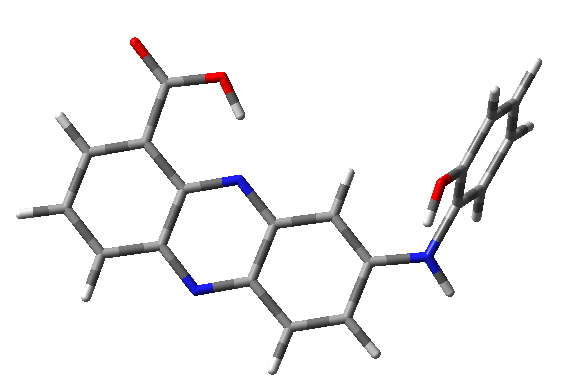** | -1121.5964619 | -703812.99580687 | 26.64 |
| **2-1c** | **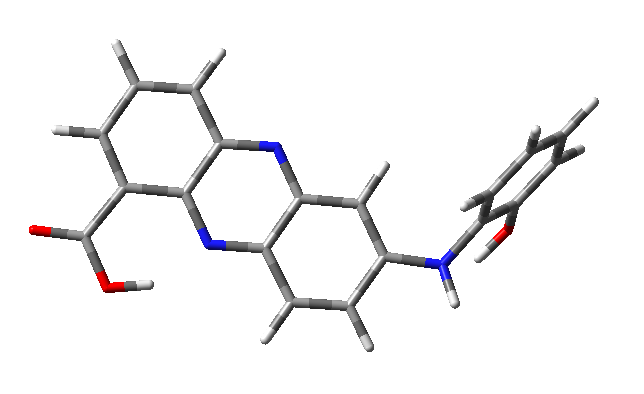** | -1121.59646185 | -703812.99577549 | 26.73 |
| **2-1d** | **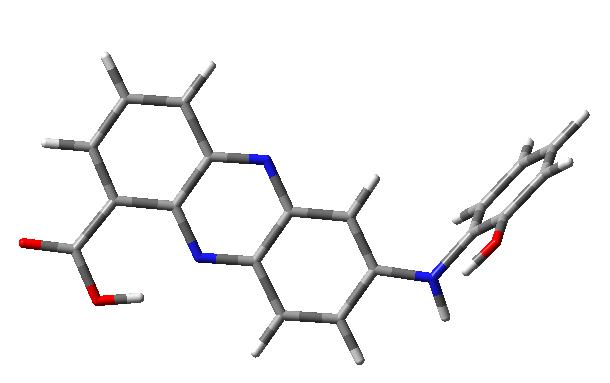** | -1121.5964617 | -703812.99568137 | 26.72 |
| **2-1e** | **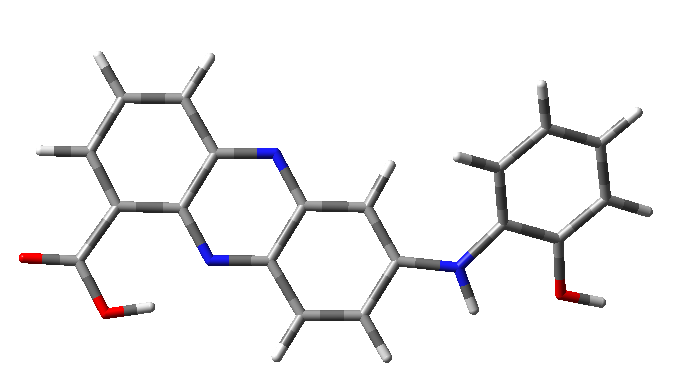** | -1121.59645878 | -703812.99384904 | 26.64 |
| **2-1f** | **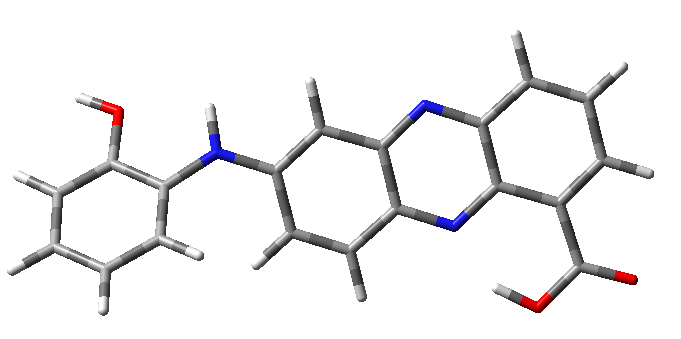** | -1121.59509015 | -703812.13502003 | 6.25 |
| **2-2g** | **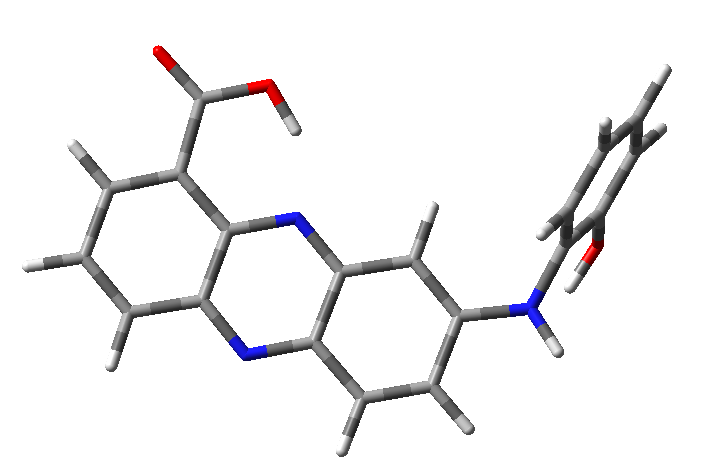** | -1121.5980202 | -703813.97365570 | 43.32 |
| **2-2h** | **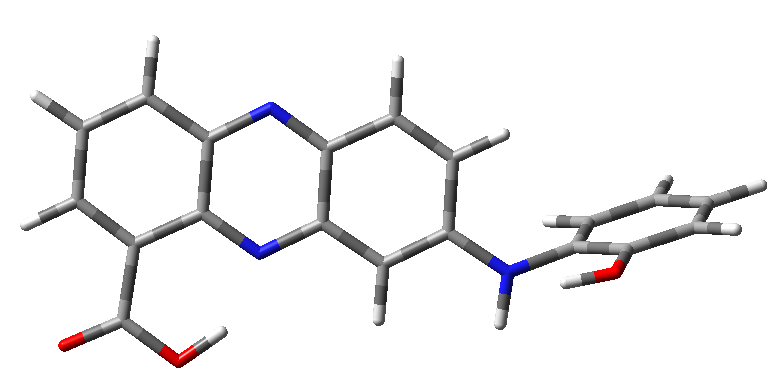** | -1121.59735699 | -703813.55748480 | 21.46 |
| **2-2i** | **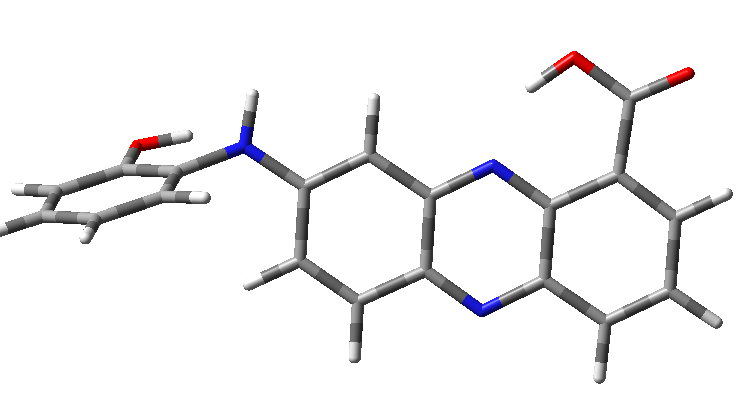** | -1121.59735827 | -703813.55828801 | 21.49 |
| **2-2j** | **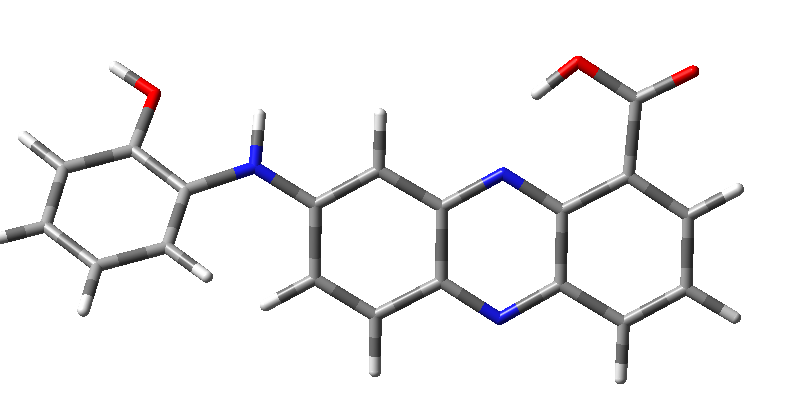** | -1121.59693496 | -703813.29265675 | 13.72 |

**Table S4** DP4+ analysis of calculated ^13^C-NMR data of **2**


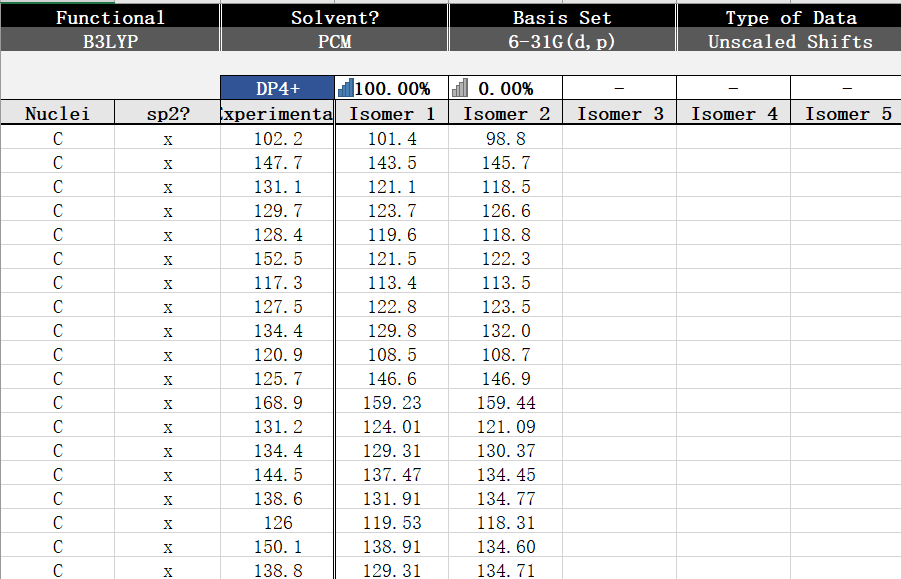


**Figure S1**. The ^1^H NMR spectrum of compound **1** in CD_3_OD

**Figure S2**. The ^13^C NMR spectrum of compound **1** in CD_3_OD

**Figure S3**. The HMQC spectrum of compound **1** in CD_3_OD

**Figure S4**. The HMQC expanded regions of compound **1** in CD_3_OD

**Figure S5**. The ^1^H-^1^H COSY spectrum of compound **1** in CD_3_OD

**Figure S6**. The ^1^H-^1^H COSY expanded regions of compound **1** in CD_3_OD

**Figure S7**. The HMBC spectrum of compound **1** in CD_3_OD

**Figure S8**. The HMBC expanded regions of compound **1** in CD_3_OD

**Figure S9**. The HRESIMS spectrum of compound **1**


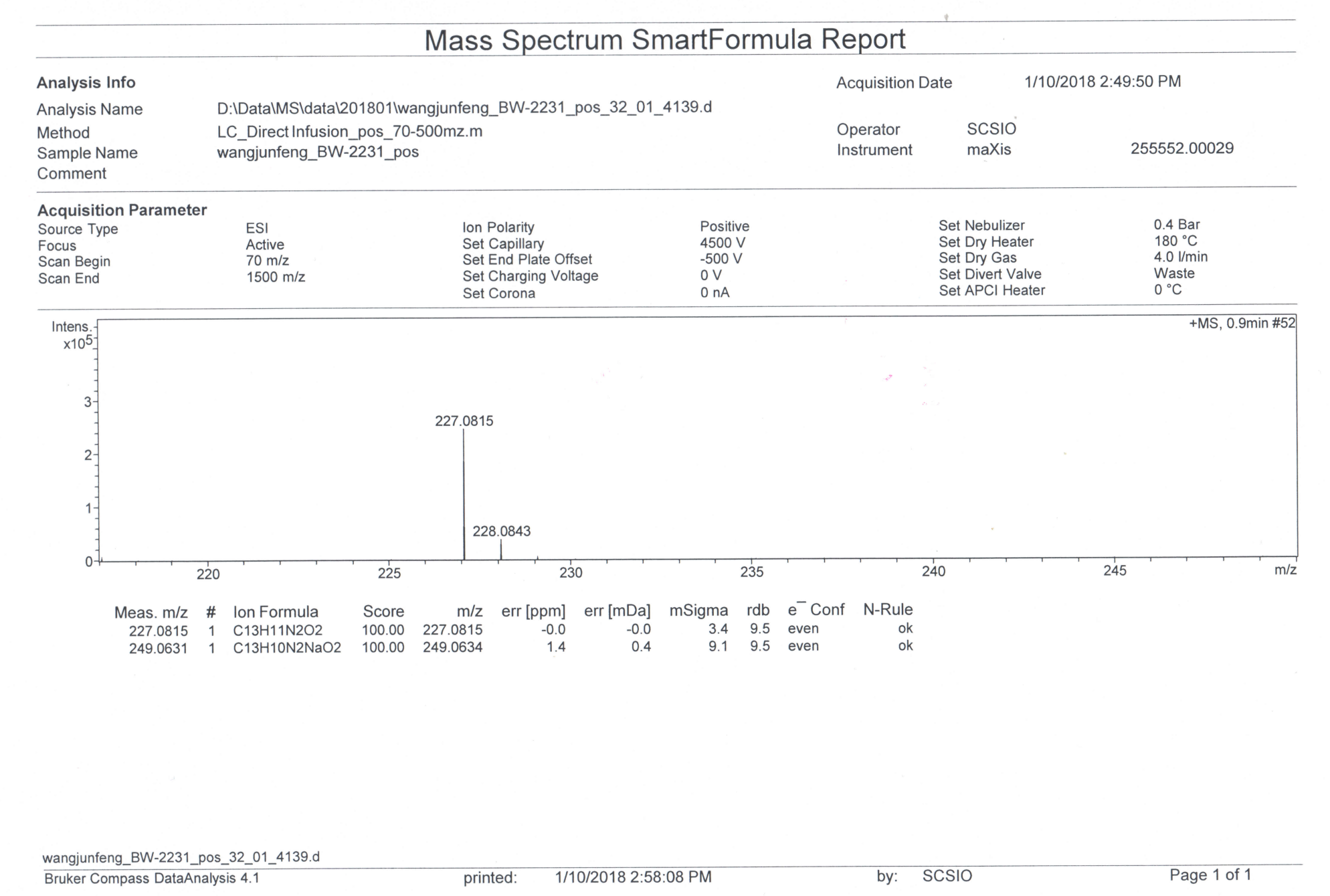


**Figure S10**. The UV spectrum of compound **1**

**Figure S11**. The ^1^H NMR spectrum of compound **2** in CD_3_OD

**Figure S12**. The ^13^C NMR spectrum of compound **2** in CD_3_OD

**Figure S13**. The HMQC spectrum of compound **2** in CD_3_OD

**Figure S14**. The HMQC expanded regions of compound **2** in CD_3_OD

**Figure S15**. The ^1^H-^1^H COSY spectrum of compound **2** in CD_3_OD

**Figure S16**. The ^1^H-^1^H COSY expanded regions of compound **2** in CD_3_OD

**Figure S17**. The HMBC spectrum of compound **2** in CD_3_OD

**Figure S18**. The HMBC expanded regions of compound **2** in CD_3_OD

**Figure S19**. The HRESIMS spectrum of compound **2**


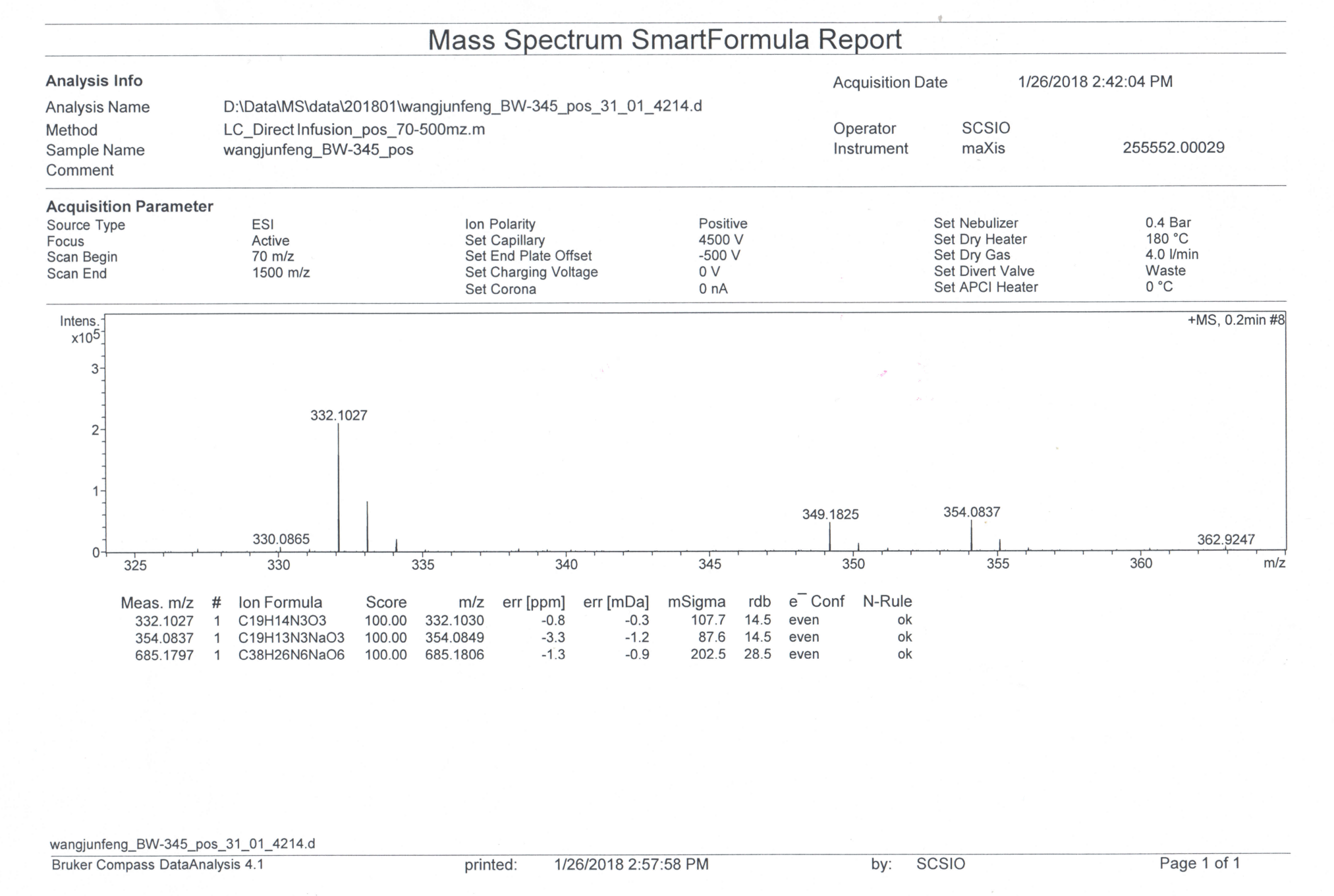


**Figure S20**. The UV spectrum of compound **2**

**Figure S21**. The ^1^H NMR spectrum of compound **3** in DMSO-*d*_6_

**Figure S22**. The ^13^C NMR spectrum of compound **3** in DMSO-*d*_6_

**Figure S23**. The HMQC spectrum of compound **3** in DMSO-*d*_6_

**Figure S24**. The ^1^H-^1^H COSY spectrum of compound **3** in DMSO-*d*_6_

**Figure S25**. The HMBC spectrum of compound **3** in DMSO-*d*_6_

**Figure S26**. The ROESY spectrum of compound **3** in DMSO-*d*_6_


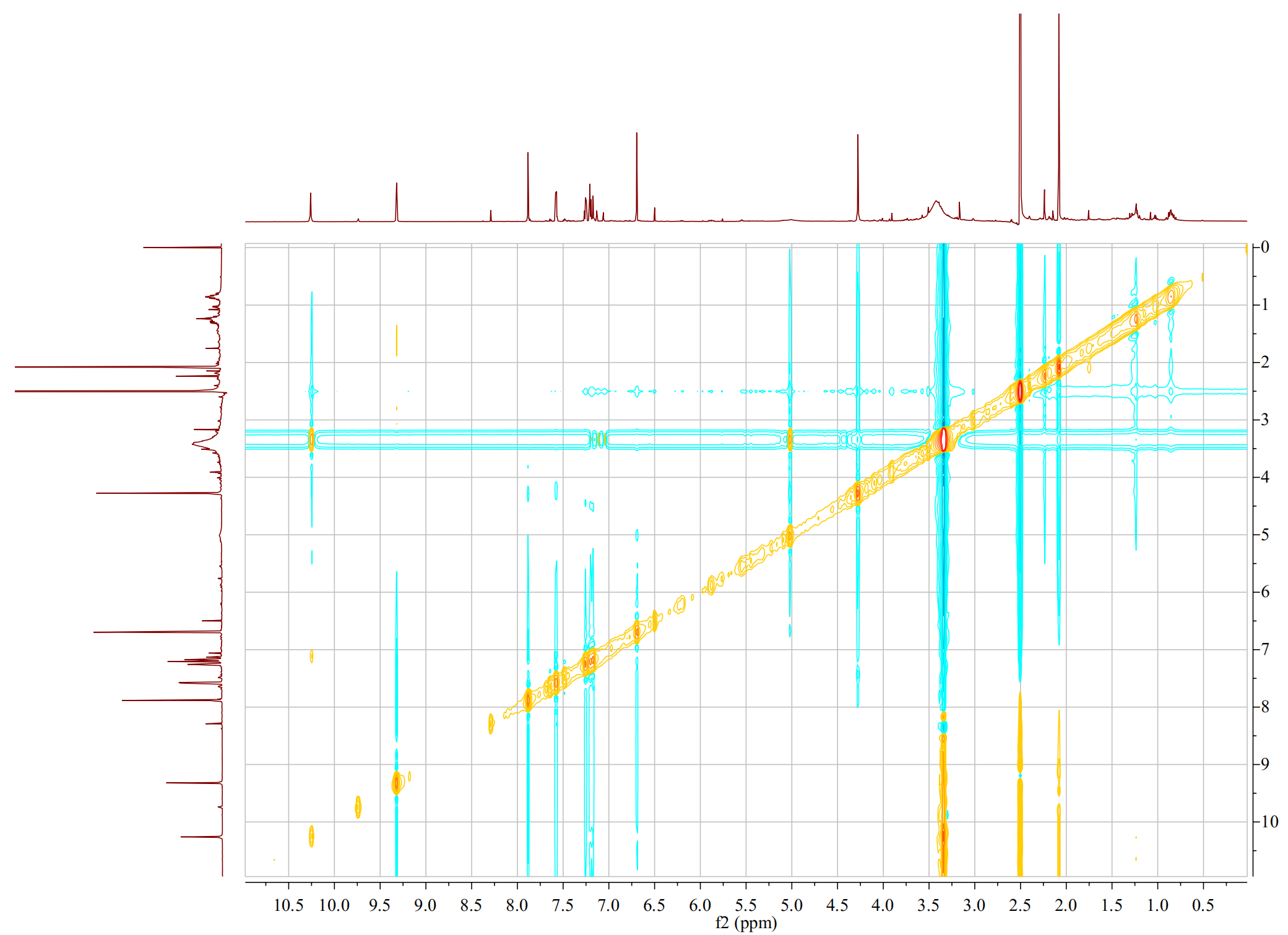


**Figure S27**. The HRESIMS spectrum of compound **3**


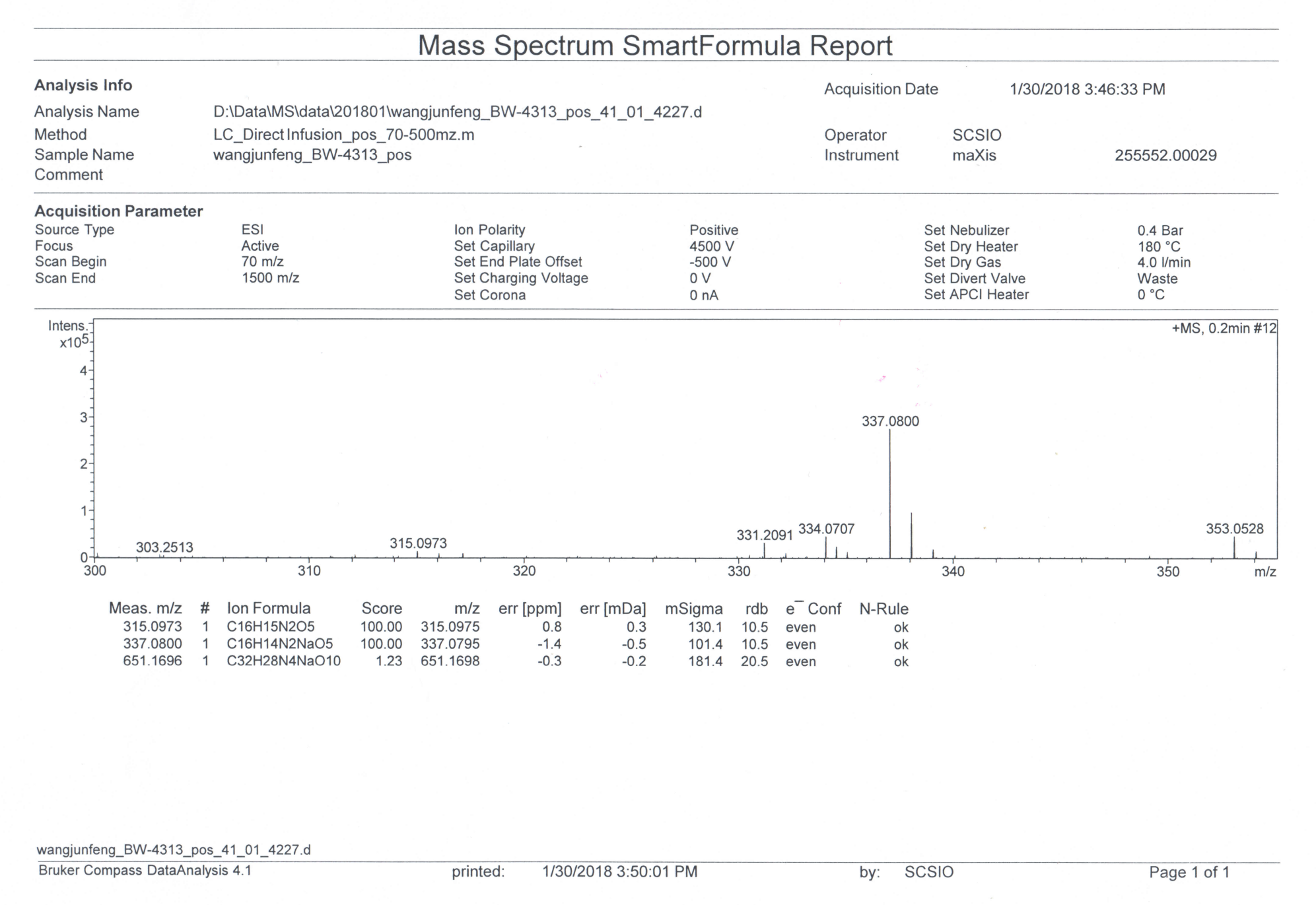


**Figure S28**. The UV spectrum of compound **3**
